# The Safety Properties of a Potential Kind of Novel Green Primary Explosive: Al/Fe_2_O_3_/RDX Nanocomposite

**DOI:** 10.3390/ma11101930

**Published:** 2018-10-10

**Authors:** Qingping Luo, Xinping Long, Fude Nie, Guixiang Liu, Mingshui Zhu

**Affiliations:** 1 State Key Laboratory of Environmental-Friendly Energy Materials, Southwest University of Science and Technology, Mianyang 621010, China; luoqingping74@126.com (Q.L.); liuguuixuang79@126.com (G.L.); 2 Institute of Chemical Materials, Chinese Academy of Engineering Physics (CAEP), Mianyang 621900, China; zgh-nfd@sohu.com (F.N.); zhumingshui@163.com (M.Z.)

**Keywords:** green primary explosives, Al/Fe_2_O_3_/RDX nanocomposite, sensitivity

## Abstract

Green primary explosives have gained wide attention for environmental protection. A potential novel lead-free primary explosive, Al/Fe_2_O_3_/RDX hybrid nanocomposite was prepared by ultrasonic mixing, and its safety properties are discussed in detail. Results showed that their sensitivity and safety properties were a function of the specific surface area and proportions of their ingredients. Their impact sensitivity fell and their static discharge, flame, and hot bridge wire sensitivities rose as the specific surface area of nano-Fe_2_O_3_ increased. As the amount of Al/Fe_2_O_3_ nanothermite was increased, its impact sensitivity fell and its flame sensitivity rose; their static discharge and hot bridge wire sensitivities, however, followed an inverted “U” type change trend and were determined by both the particle size of the ingredients and the resistance of the nanocomposite. Their firing properties in an electric detonator depended on the proportion of the constituents. Thus, green nanoscale primary explosives are appropriate for a range of initiatory applications and can be created by adjusting their specific surface area and the amount of their constituents.

## 1. Introduction

Initiating explosives are sensitive explosives widely used in military munitions and civilian applications that deflagrate or detonate with low external stimuli, e.g., a flame, pinprick, friction, static discharge, or moderate heat, and release enough energy to initiate other explosives. Traditional initiating explosives, that contain toxic metallic elements such as mercury fulminate, lead azide, or lead styphnate, have inherent drawbacks, such as hydrolytic instability or environmental pollution, and so, their military and civilian uses are limited for the sake of environmental protection [1,2,3]. Consequently, new green initiating explosives have attracted much attention from researchers in the field of primary explosives [4,5]. Some studies have explored particular green initiating explosives, such as tetrazoles, and their derivatives, furazans [6,7,8,9,10,11,12,13]. However, their synthesis is very complicated, and their production generates pollutants [6,8,10,11,12,13]. In addition, in the past decade some nano-energetic materials (for example, metastable intermolecular composites, MIC), with the potential to be used as primary explosives, have also excited great interest from military experts [14,15].

Due to their remarkable ignition and energy release properties, one of the nano-energetic materials, nanothermite, has been gaining increasing attention from researchers in the fields of propellants, explosives, and pyrotechnics [16,17,18,19,20]. Nanothermites, however, do not produce gas during the thermite reaction and they cannot self-detonate, which reduces their usefulness in military and civilian industry. Secondary explosives like hexogen (RDX) or cyclotetramethylenete-tranitramine (HMX) do evolve massive amounts of gas during their chemical reactions, but their volume energy densities are lower than those of nanothermites. Therefore, it appears that nanothermites, mixed with secondary explosives, might perform better than either nanothermites or secondary explosives. Based on this idea, some studies of hybrid nanocomposites have been conducted [21,22,23], and their combustion characteristics show that they could cause a rapid deflagration-to-detonation transition (DDT) that can be accelerated to the primary explosive level [22] and therefore used to initiate the detonation of a high explosive [23]. In addition, their preparation, by mixing, is simple and generates no pollutants. Therefore, these explosives might be a kind of novel green nanoscale substitute for existing initiating explosives, thanks to their high thermal stabilities and controllable velocities of detonation. Furthermore, due to the absence of lead, and if used as primary explosives, they would escape some of the shortcomings of current primary explosives.

The sensitivity of initiating explosives to external stimuli makes them a potential safety hazard. It is for this reason that their safety properties are crucial to their use in both military munitions and civilian industry. Few systematic evaluations of the safety properties of hybrid nanocomposites have been conducted, however. In this paper, the safety properties of Al/Fe_2_O_3_/RDX hybrid nanocomposites, a novel lead-free nanoscale initiatory compound prepared by mixing Al/Fe_2_O_3_ nanothermite with RDX, are studied in detail.

## 2. Materials and Methods

### 2.1. Preparation of the Materials

**Figure 1:**
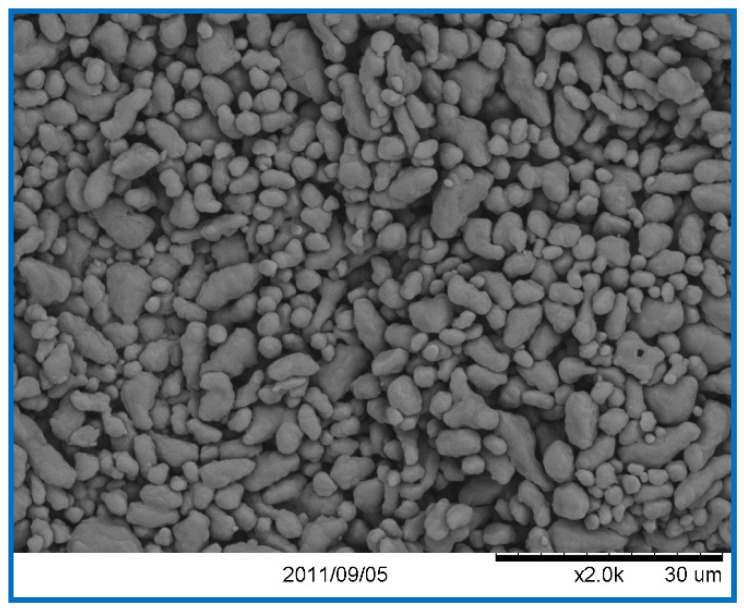
SEM picture of super fine RDX.

**Figure 2:**
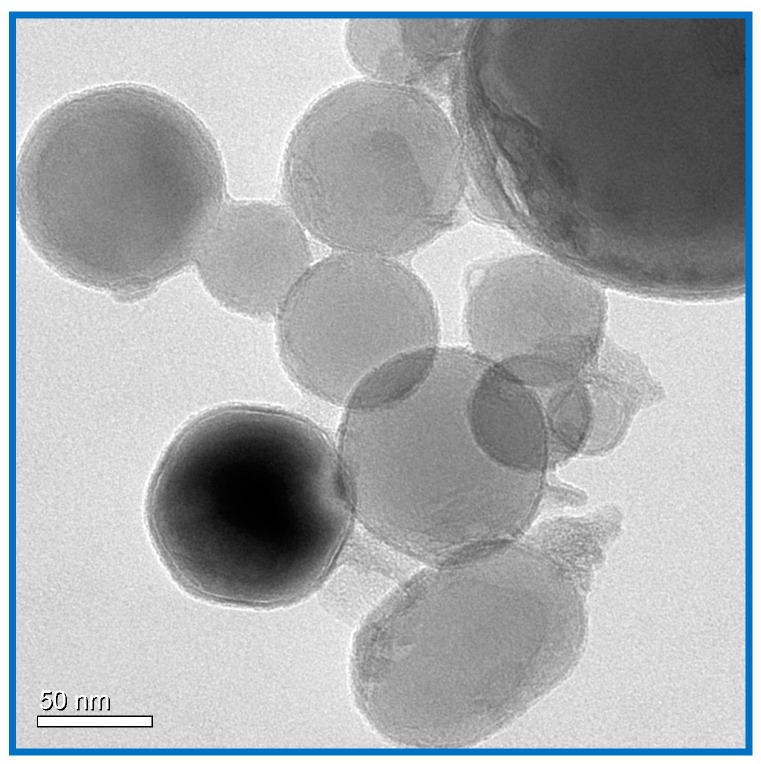
TEM picture of nano-Al.

Superfine RDX with a particle size of 1–5 μm was prepared in our lab by spraying RDX solution into deionized water at high-pressure and then recrystallized and freeze-dried. The result of this is shown in Figure 1. Nano-Fe_2_O_3_, with specific surface areas of 10.3 m^2^/g, 43.2 m^2^/g, and 230 m^2^/g, was synthesized by the sol-gel method and by a low temperature CO_2_ supercritical process as described by Luo et al. [24]. The specific surface area was measured using an American Quantachrome ADSORP gas adsorption analyzer and BET theory and five data points between relative pressures 0.05 and 0.3 at 77 K [25]. Passivated nano-Al powder, with an average particle size of 40 nm, a ~5 nm oxide shell, and an active metal content of 52 wt % was obtained from a commercial technology company (Figure 2). The Al/Fe_2_O_3_/RDX nanocomposites were prepared by mixing RDX with Al/Fe_2_O_3_ nanothermite composed of nano-Fe_2_O_3_ and nano-Al powders according to Equation (1) and an equivalence ratio Φ of 1.1 (Al-rich) in the solvent cyclohexane or cyclohexane plus acetone. The resulting mixtures were treated using an ultrasonification process for 3 h to ensure more homogeneous mixing. Al/Fe_2_O_3_/RDX hybrid nanocomposites were then obtained by drying at 55 °C for 48 h in a vacuum drying cabinet to evaporate the solvents. The prepared nanocomposites were labelled with their RDX content (for example, 30 wt % (Al/Fe_2_O_3_)/70 wt % RDX = R − 70).

(1) Φ=(active AlFe2O3)Actual(active AlFe2O3)Stoichiometric

### 2.2. Sensitivity Tests of the Al/Fe_2_O_3_/RDX Nanocomposites

The impact sensitivities of the Al/Fe_2_O_3_/RDX nanocomposites were determined by the Fall Hammer Method using a 2.0 kg drop weight, 25 cm height, and 30 mg quantity. Their friction sensitivities were assessed using a 1.5 kg hammer weight, a 66° switch angle, 2.45 MPa, and a 20 mg quantity. Twenty-five samples were tested, and the firing percent was calculated for the various impact and friction sensitivities. The static discharge sensitivity of each sample was measured by an electric spark sensitivity analyzer with an electrode gap of 0.5 mm, an electrical capacity of 30.5 kpF, and a 20 mg quantity. The firing energy E_50_ was calculated using the formula, E_50_ = 0.5C(V_50_)^2^, where V_50_ is the 50% firing voltage and C is the electrical capacity of the electrode [26]. The flame sensitivity of each sample was determined using the fuse method with a fuse length of 7 cm and a 20 mg quantity, and the jetting distance of the fuse flame at 50% firing was made for flame sensitivity [27]. For the safety evaluation, 0.1 g of Al/Fe_2_O_3_/RDX nanocomposite was placed in an electric detonator with a diameter of 6.3 mm and pressed at 802 kPa; the loading density was 0.48 g/cm^3^. The electrostatic safety of each sample was measured at 25 kV/500 pF/5 kΩ, its hot bridge wire sensitivity was measured by heating the bridge wire at different voltages to simulate firing conditions in an electric detonator, and the firing energy was calculated using the formula, *E* = *I*^2^*Rt*, where *I* is the intensity of the current, *R* is the electrical resistance of the hot bridge wire, and *t* is the firing time.

## 3. Results and Discussion

### 3.1. Mechanical Sensitivity Analysis

The mechanical sensitivity of Al/Fe_2_O_3_/RDX nanocomposites containing various amounts of RDX and nano-Fe_2_O_3_ with various specific surface areas were measured using the test criteria for high-sensitivity explosives. The results are given in Table 1.

As Table 1 shows, the mechanical sensitivity of Al/Fe_2_O_3_/RDX nanocomposites depended heavily on their RDX content and the specific surface area of the nano-Fe_2_O_3_ in them. Their impact sensitivity decreased slightly as their RDX content decreased and also decreased as the specific surface area of the nano-Fe_2_O_3_ in them increased. In the light of hot spot theory, this may be due to the nano-Fe_2_O_3_ particles with low specific surface areas and large particle sizes forming local-center hot-stress regions at impact, which would readily cause the nanocomposites to fire, thereby increasing their impact sensitivity. The friction sensitivity of nanocomposites increased dramatically and then remained the same once Al/Fe_2_O_3_ nanothermite was added to RDX. This might be due to breakage of the Al_2_O_3_ crust on the surface of the nano-Al particles at the blade load, bringing highly reactive nano-Al into direct contact with nano-Fe_2_O_3_, with the resulting thermite reaction between nano-Al and nano-Fe_2_O_3_ releasing a large amount of heat, and forming a partial high-temperature zone, which accelerated the thermite reaction and the decomposition of RDX, leading to the firing of the Al/Fe_2_O_3_/RDX nanocomposite. Therefore, the nanocomposites had high friction sensitivity, much higher than that of RDX. A loud explosion and a geyser of sparks resulted when testing the samples for friction sensitivity. However, it is difficult to break the Al_2_O_3_ crust of nano-Al particles at the percussive force. On the contrary, some of the impact energy was absorbed by the Al and Fe_2_O_3_ particles, causing the nanocomposites to be much more sensitive to friction than to impact.

### 3.2. Static Discharges Sensitivity Analysis

The static discharge sensitivity of Al/Fe_2_O_3_/RDX nanocomposites was measured at voltage (V_50_) and firing energy (E_50_) at 50% firing when the samples were put between two electrodes. The static discharge sensitivity of nanocomposites with different RDX content, specific surface areas of nano-Fe_2_O_3_, and solvents used in the mixing process are given in Table 2.

As Table 2 shows, the RDX content, the specific surface area of Fe_2_O_3_, and the solvent used in the mixing process greatly affected the V_50_ and E_50_ of Al/Fe_2_O_3_/RDX nanocomposites. The V_50_ and E_50_—i.e., the static discharge sensitivities of most of the Al/Fe_2_O_3_/RDX nanocomposites were much lower than that of pure RDX or Al/Fe_2_O_3_ nanothermite. As the amount of Al/Fe_2_O_3_ nanothermite increased, the static discharge sensitivity of the nanocomposites first increased, however, and then decreased, following an inverted “U” type change trend. This may be due to changes in particle size and resistance when Al/Fe_2_O_3_ nanothermite was added to RDX [28,29]. As the amount of Al/Fe_2_O_3_ nanothermite increased, the average particle size of the nanocomposite fell and the specific surface area increased, adding to the effective friction area and thereby making electric charges accumulate easily on the surfaces of the nanocomposites, leading to firing at a low electrostatic voltage [30]. In addition, the increased amount of Al/Fe_2_O_3_ nanothermite reduced the resistance of the nanocomposite due to the metal, aluminum, being a conductor. The resistance of Al/Fe_2_O_3_ nanothermite was far less than that of RDX, and so electrical charges accumulated less on its surfaces, increasing its firing energy and decreasing its static discharge sensitivity. The inverted “U” type change trend for the static discharge sensitivity of the nanocomposites was the result of the interaction of the particle sizes and the resistance of their ingredients.

The V_50_ and E_50_ for the nanocomposites decreased as the specific surface area of the nano-Fe_2_O_3_ increased, and their V_50_ and E_50_ also decreased when both cyclohexane and acetone were used instead of cyclohexane alone. This may be because the effective friction area increased with the increase in the specific surface area, causing more static discharges to accumulate on the surfaces of the sample, which resulted in a decrease in the sample’s V_50_ and E_50_ and an increase in its static discharge sensitivity. When cyclohexane plus acetone was used as the solvent, the average particle size in the resulting nanocomposite was smaller than when cyclohexane alone was used, leading to an increase in static discharge sensitivity.

**Figure 3:**
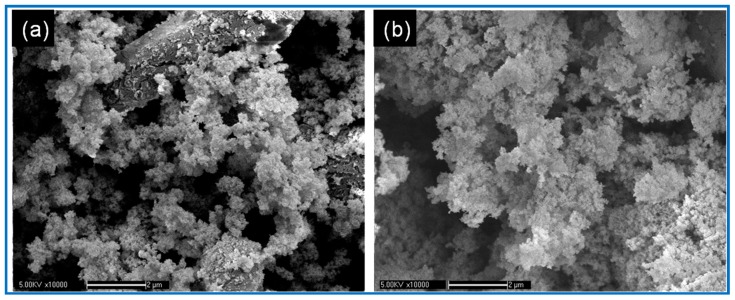
SEM images of Al/Fe_2_O_3_/RDX nanocomposites prepared using (**a**) cyclohexane or (**b**) both cyclohexane and acetone.

Figure 3 gives SEM images of Al/Fe_2_O_3_/RDX nanocomposites prepared using different solvents. As shown in Figure 3a, the use of cyclohexane produced large, 1 μm particles and small, <100 nm particles. The large particles are super fine RDX, and the small particles are nano-Al/Fe_2_O_3_ composite. When both cyclohexane and acetone were used, the particle sizes of the nanocomposite were less than 100 nm, and the granularity distribution was more uniform because RDX dissolves in acetone. Therefore, a hybrid nanocomposite prepared using both cyclohexane and acetone will have smaller RDX particles and a higher static discharge sensitivity than nanocomposite prepared using cyclohexane alone. Nanocomposite’s static discharge sensitivity was greatest when the amount of Al/Fe_2_O_3_ nanothermite was 50 wt % and both cyclohexane and acetone were used. Thus, Al/Fe_2_O_3_/RDX nanocomposites with different static discharge sensitivities can be produced by adjusting the RDX content and the particle sizes of the constituents.

### 3.3. Flame Sensitivity Analysis

As stated, the flame sensitivity of Al/Fe_2_O_3_/RDX nanocomposites was determined using the fuse method. The results are given in Table 3.

As shown in Table 3, superfine RDX was very stable and did not fire even when the fuse’s combustion flame was as close as 1.2 mm, indicating that the flame sensitivity of super fine RDX is very low. As the proportion of Al/Fe_2_O_3_ nanothermite increased, the ignition distance and the flame sensitivity of the nanocomposites both also increased—i.e., the larger the amount of Al/Fe_2_O_3_ nanothermite, the longer the ignition distance of the Al/Fe_2_O_3_/RDX nanocomposite, and the greater its flame sensitivity. This may be due to the good thermal conductivity of the nano-Al and the high reactivity of the Al/Fe_2_O_3_ nanothermite in the nanocomposite. When the fuse’s combustion flame contacted the surface of the nanocomposite, the nano-Al on the surface of the nanocomposite presumably adsorbed enough heat from the flame to make the Al/Fe_2_O_3_ nanothermite react, releasing abundant heat that formed a local high-temperature region in which the surface of the RDX generated a hot spot that made the RDX decompose. It was this process that produced the voluminous smoke that was seen during testing. The Al/Fe_2_O_3_ nanothermite around the high-temperature region also fired, and finally the nanocomposite fired as a whole.

Due to this chain of events, hybrid nanocomposites containing Al/Fe_2_O_3_ nanothermite were more sensitive to heat than RDX, and the nanocomposites containing a large amount of Al/Fe_2_O_3_ nanothermite showed high flame sensitivity. As the specific surface area of Fe_2_O_3_ increased, the firing distance and flame sensitivity of the Al/Fe_2_O_3_/RDX nanocomposite also increased. If both cyclohexane and acetone were used as the solvent during mixing, the flame sensitivity of the nanocomposite also increased because RDX dissolves in the acetone, reducing its particle size and increasing the specific surface area of the particles. Thus, the particle size of the ingredients in the nanocomposites had a large effect on their flame sensitivity.

### 3.4. Hot Bridge Wire Sensitivity Analysis

**Figure 4:**
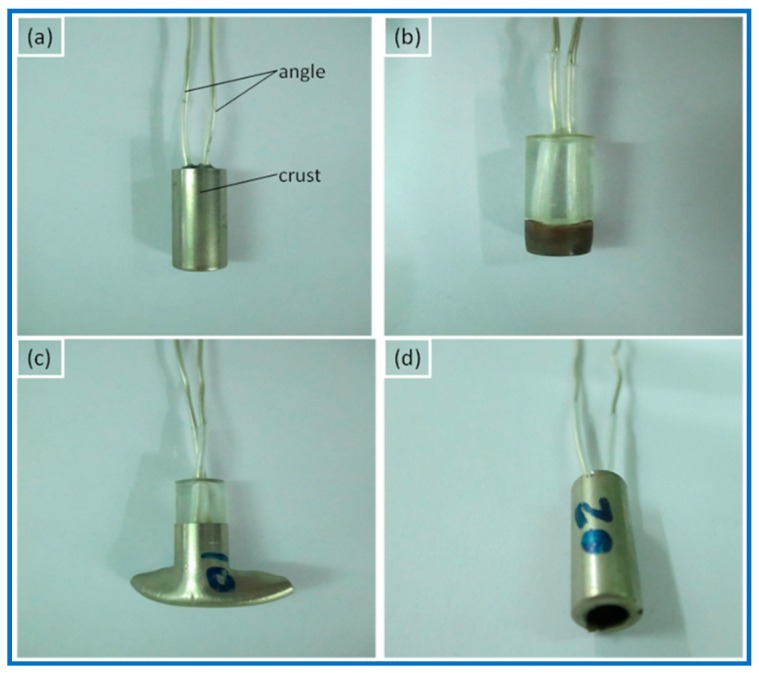
Firing pictures at the angle–angle connection for Al/Fe_2_O_3_/RDX nanocomposites with RDX content of (**a**) R-100, (**b**) >50 wt % RDX, (**c**) R-30, (**d**) R-0.

The static discharge safety properties of Al/Fe_2_O_3_/RDX nanocomposites in an electric detonator were measured using the angle–crust connection mode and the angle–angle connection mode. The results are given in Table 4. The firing pictures of nanocomposites with different RDX content at the angle–angle connection mode are shown in Figure 4.

As Table 4 shows, pure RDX in an electric detonator was very stable and did not fire at the angle–crust connection or at the angle–angle connection in the static discharge safety test (Figure 4a). In contrast, the different connection circuit modes considerably affected the static discharge safety properties of Al/Fe_2_O_3_/RDX nanocomposites in electric detonators. The fact that the nanocomposites did not fire completely at the angle–crust connection may be due to the good conductivity of the metal crust of the electric detonator, which impeded electrostatic accumulation on its surface. As a result, the nanocomposites did not fire at the angle–crust connection, indicating that they were very safe in that connection mode. They did fire at the angle–angle connection (Figure 4), however, with their firing strength decreasing as the proportion of RDX decreased. Some nanocomposites with a high RDX content (>50 wt % RDX) exploded in the static discharge safety test, and the metal crusts of the electric detonators disappeared (Figure 4b). When the RDX content was 30 wt % (Figure 4c), the nanocomposite also exploded, splitting and deforming the metal crusts. Al/Fe_2_O_3_ nanothermite also fired at the angle–angle connection, but only combustion occurred, and the metal crust was only burned through, not destroyed or deformed (Figure 4d). The specific surface area and the solvent used during mixing had little effect on the static discharge safety properties of the nanocomposites in an electric detonator. Different firing properties were achieved by adjusting the proportion of RDX in the nanocomposites.

The hot bridge wire sensitivity of Al/Fe_2_O_3_/RDX nanocomposites was measured by determining the firing properties of nanocomposites with different specific surface areas of Fe_2_O_3_, solvents used in mixing, and RDX content through heating the bridge wire at 5 A in an electric detonator. The results are given in Table 5.

As Table 5 shows, pure RDX was very stable and did not fire when the bridge wire was heated at 5 A, but as Al/Fe_2_O_3_ nanothermite was added to the RDX, the resulting nanocomposite did fire at that amperage. This is because the addition of Al/Fe_2_O_3_ nanothermite improved the firing properties of RDX. As the proportion of RDX decreased, the firing energy of the nanocomposites first decreased and then increased, following a “U” type change trend that is the reverse of the change trend for their hot bridge wire sensitivity. The increase in the specific surface area of Fe_2_O_3_ and the use of both cyclohexane and acetone increased their hot bridge wire sensitivity, because these two factors increased the specific surface area of the nanocomposites, leading to an increase in their reactivity and a decrease in their firing energy. The nanocomposite had the lowest firing energy (87.9 mJ) when the proportion of Al/Fe_2_O_3_ nanothermite was 50 wt %, and both cyclohexane and acetone were used as the solvent. The hot bridge wire sensitivity of the nanocomposites depended heavily on their RDX content and specific surface area.

## 4. Conclusions

The safety properties of one potential kind of novel green nanoscale primary explosive were evaluated by measuring its sensitivities. These were greatly affected by the RDX content and the particle sizes of its ingredients, and they were heavily dependent on its specific surface area and its ingredients. As the specific surface area of nano-Fe_2_O_3_ increased, the impact sensitivity of Al/Fe_2_O_3_/RDX nanocomposite fell, but the static discharge, flame, and hot bridge wire sensitivities all increased. When both cyclohexane and acetone were used as the solvent during mixing, the sensitivity of the nanocomposite also increased, due to the decrease in the RDX particle size. As the content of Al/Fe_2_O_3_ nanothermite increased, impact sensitivity fell, and flame sensitivity rose. However, the static discharge and hot bridge wire sensitivities of the nanocomposite followed an inverted “U” type change trend and were determined by both the particle size of the ingredients and the resistance of the nanocomposite. Nanoscale primary explosives with different sensitivities and firing properties can be obtained by adjusting their RDX content and the specific surface area of their ingredients.

## Figures and Tables

**Table 1:** Mechanical sensitivities of Al/Fe_2_O_3_/RDX nanocomposites.

Sample	Specific Surface Area of Fe_2_O_3_ (m^2^/g)	Impact Sensitivity (%)	Friction Sensitivity (%)
R-100	-	8	8
R-70	43.2	16	100
R-70	230	12	100
R-50	10.3	16	100
R-50	43.2	12	100
R-50	230	8	100
R-30	43.2	8	100
R-30	230	8	100
R-0	230	0	100

**Table 2:** Static discharge sensitivity of Al/Fe_2_O_3_/RDX nanocomposites.

Sample	Specific Surface Area of Fe_2_O_3_ (m^2^/g)	Solvent	V_50_ (kV)	E_50_ (mJ)
R-100	-	-	4.824	355.1
R-70	43.2	cyclohexane	3.367	173
R-70	230	cyclohexane	3.1	147
R-70	230	acetone + cyclohexane	2.5	95.3
R-50	230	cyclohexane	1.9	55.05
R-50	230	acetone + cyclohexane	<1.8	<49.41
R-30	230	acetone + cyclohexane	2.8	119.6
R-0	230	cyclohexane	6.04	560

**Table 3:** Flame sensitivity of Al/Fe_2_O_3_/RDX nanocomposites.

Sample	Specific Surface Area of Fe_2_O_3_ (m^2^/g)	Solvent	Ignition Distance (mm)
R-100	-	-	<1.2 mm
R-70	43.2	Cyclohexane	9.0
R-70	230	Cyclohexane	15
R-70	230	acetone + cyclohexane	30
R-50	230	Cyclohexane	35
R-50	230	acetone + cyclohexane	70
R-30	230	acetone + cyclohexane	>80
R-0	230	Cyclohexane	>80

**Table 4:** Static discharge safety properties of Al/Fe_2_O_3_/RDX nanocomposites in an electric detonator.

Sample	Specific Surface Area of Fe_2_O_3_ (m^2^/g)	Solvent	Firing Property at Static Discharge	
			Angle–Crust	Angle–Angle
R-100	-	-	no	no
R-70/50/30/0	10.3/43.2/230	cyclohexane/acetone + cyclohexane	no	yes

**Table 5:** Hot bridge wire sensitivities of Al/Fe_2_O_3_/RDX nanocomposites.

Sample	Specific Surface Area of Fe_2_O_3_ (m^2^/g)	Solvent	Firing Conditions	Electrical Resistance of Bridge Wire (Ω)	Firing Energy (mJ)
R-100	-	-	-	0.995	-
R-70	43.2	cyclohexane	5 A/14.8 ms	1.082	400.3
R-70	230	cyclohexane	5 A/10.2 ms	0.965	245.3
R-70	230	Acetone + cyclohexane	5 A/4.5 ms	1.001	112.6
R-50	230	cyclohexane	5 A/3.9 ms	1.040	101.4
R-50	230	Acetone + cyclohexane	5 A/3.5 ms	1.005	87.9
R-30	230	cyclohexane	5 A/9.3 ms	0.975	226.7
R-30	230	Acetone + cyclohexane	5 A/6.6 ms	0.946	156.1
R-0	230	cyclohexane	5 A/17.8 ms	1.002	445.9
